# The Impact of Biomarkers on the Early Detection of Acute Mesenteric Ischemia

**DOI:** 10.3390/biomedicines12010085

**Published:** 2023-12-29

**Authors:** Aleksandar Zafirovski, Marija Zafirovska, Dimitrij Kuhelj, Tadeja Pintar

**Affiliations:** 1 Faculty of Medicine, University of Ljubljana, Vrazov trg 2, 1000 Ljubljana, Slovenia; aleksandarzafirovski5@gmail.com (A.Z.); m.zafirovska27@gmail.com (M.Z.); dimitrij.kuhelj@guest.arnes.si (D.K.); 2 Department of Radiology, General Hospital Jesenice, Cesta Maršala Tita 112, 4270 Jesenice, Slovenia; 3 Clinical Institute of Radiology, University Medical Centre Ljubljana, Zaloška cesta 7, 1000 Ljubljana, Slovenia; 4 Department of Abdominal Surgery, University Medical Centre Ljubljana, Zaloška cesta 2, 1000 Ljubljana, Slovenia

**Keywords:** biomarkers, acute mesenteric ischemia, early diagnosis

## Abstract

Background: acute mesenteric ischemia (AMI) is a life-threatening condition that is caused by inadequate blood flow through the mesenteric vessel and is related to high mortality rates due to systemic complications. This study aims to systematically review the available literature concerning the major findings of possible biomarkers for early detection of acute mesenteric ischemia in the human population. Methods: studies that measured the performance of biomarkers during acute mesenteric ischemia were identified with the search of PubMed, Embase, Medline, and Cochrane library. Results: from a total of 654 articles, 46 articles examining 14 different biomarkers were filtered, falling within our inclusion criteria. Intestinal fatty acid-binding protein (I-FABP) was the most commonly researched biomarker regarding AMI, with sensitivity ranging from 61.5% to 100% and specificity ranging from 40% to 100%. The second most commonly researched biomarker was D-dimer, with a sensitivity of 60–100% and a specificity of 18–85.71%. L-lactate had a sensitivity of 36.6–90.91% and a specificity of 64.29–96%. Several parameters within the blood count were examined as potential markers for AMI, including NLR, PLR, MPV, RDW, DNI, and IG. Citrulline, interleukin 6 (IL-6), and procalcitonin (PCT) were the least-researched biomarkers. Conclusion: different biomarkers showed different accuracies in detecting AMI. I-FABP and D-dimer have been the most researched and shown to be valuable in the diagnosis of AMI, whereas L-lactate could be used as an additional tool. Ischemia-modified albumin (IMA), alpha glutathione S-transferase (αGST), interleukin 6 (IL-6), and citrulline showed potential use in their respective studies. However, further research needs to be done on larger sample sizes and with controls to reduce bias. Several studies showed that neutrophil–lymphocyte ratio (NLR), platelet–lymphocyte ratio (PLR), mean platelet volume (MPV), red-cell distribution width (RDW), delta neutrophil index (DNI), and immature granulocytes (IGs) might be useful, as well at the same time be widely distributed and affordable in combination with other markers presenting higher specificity and sensitivity.

## 1. Introduction

Acute mesenteric ischemia (AMI) is a life-threatening condition characterized by inadequate blood flow in the mesenteric vessels, leading to ischemia and eventual necrosis of the intestines. AMI encompasses four different clinical entities with different etiologies: acute mesenteric arterial embolism (AMAE), acute mesenteric arterial thrombosis (AMAT), nonocclusive mesenteric ischemia (NOMI), and mesenteric venous thrombosis (MVT) [1]. The incidence of AMI is relatively low, estimated at around 0.2% of all acute surgical admissions, but its mortality rate ranges from 50% to 80% [2]. Early and reliable detection, in addition to appropriate emergency tools, can limit the long-term consequences of AMI, which is important in terms of quality of survival and reducing the incidence of mortality.

Studies have demonstrated that intestinal ischemia occurs when the blood supply is reduced by more than 50% or the patient’s mean arterial pressure drops below 45 mmHg [3,4], although the small intestine has the ability to compensate for a reduction of up to 3⁄4 in blood supply for approximately 12 h [5]. AMI is caused by various types of pathology and its underlying factors. Conditions that lead to arterial embolism, including valvular heart disease, atrial fibrillation or flutter, myocardial infarction, cardiac valvular vegetation, mechanical valve prostheses, and cardiomyopathies lead to AMAE. AMAT can be caused by atherosclerotic disease, congestive heart failure, vasculitis, conditions that lead to low cardiac output, procoagulative status, and iatrogenic causes (cardiac catheterization-related emboli and angiography). NOMI may result from some form of shock, as can occur with heart failure, poorly controlled vasopressors, or because of excessive diuretic-associated volume depletion. On the other hand, MVT is mostly caused by conditions that lead to a hypercoagulable state, intra-abdominal infections, portal hypertension, increased intra-abdominal pressure, and venous trauma [6]. The extent of ischemic injury in the intestinal mucosa is largely reversible, except when a transmural injury occurs, leading to inflammation, necrosis, sepsis, and multiple organ failure (MOF) [7]. Symptoms of AMI are often nonspecific and include moderate to severe diffuse pain, nausea, vomiting, and diarrhea progressing to obstipation, abdominal distention, and gastrointestinal bleeding. With the progression of intestinal necrosis, signs of sepsis such as tachycardia, tachypnea, hypotension, fever, and altered mental status may develop [2].

According to the current guidelines, computed tomography angiogram (CTA) is recognized as the most efficacious diagnostic tool for detecting AMI, with a sensitivity of 85–98% and a specificity of 91–100% [1,2].

Plain abdominal radiography’s diagnostic usefulness in AMI is restricted, as it only becomes positive in cases of perforation leading to the presence of free air beneath the diaphragm. While definitive and accurate biomarkers have not been pinpointed, laboratory findings can help corroborate clinical suspicions. Potential markers like serum lactate levels, D-dimer, amylase, I-FABP, and alpha-GST could augment diagnostic precision [2]. Biomarkers have gained prominence in diverse clinical domains, assisting in disease prediction and monitoring. They offer significant advantages to both clinicians and patients by diminishing the necessity for invasive and time-consuming procedures. Furthermore, these markers can be conveniently measured in blood and urine samples [8].

This study’s objective is to systematically review the existing literature to extract crucial insights regarding potential biomarkers for the early detection of acute mesenteric ischemia in the human population.

## 2. Methods

### 2.1. Search Strategy

To identify eligible studies, a comprehensive systematic search was conducted in the PubMed, Embase, Medline, and Cochrane library databases. Two authors conducted the search of the medical literature in the period from January 2021 to April 2021, with an additional literature search performed in April 2023. 

The searched terms utilized were as follows: (exp mesenteric ischemia/OR exp intestine ischemia/OR exp mesenteric ischemia/OR acute mesenteric ischemia.mp.) AND (exp marker/OR exp biological marker/OR exp biochemical marker/OR exp molecular marker/OR exp disease marker/OR exp tumor marker/OR exp cell marker/OR cell surface marker/OR exp biopsy site marker/OR cell membrane marker/) AND (exp laboratory diagnosis/OR exp diagnosis/OR exp early diagnosis/).

Following the completion of the search, duplicate articles were removed. In the initial selection phase, two authors assessed the titles and abstracts of all identified articles based on predefined eligibility criteria. In the subsequent selection phase, full-text articles were thoroughly examined to determine their eligibility for further inclusion. Additionally, the references of relevant reviews were screened to identify any additional articles that met the inclusion criteria. In the event of any difference in opinion regarding the inclusion of an article, the authors engaged in discussions to reach a final decision. 

Our systematic review is conducted according to the Preferred Reporting Items for Systematic Reviews and Meta-Analyses (PRISMA) statement guidelines, registration number INPLASY2023120087.

### 2.2. Selection Criteria

The systematic review considered all relevant studies published in the last 30 years and written in English, Slovenian, Serbo-Croatian, and Macedonian languages. The specific criteria used for the search and selection process are shown in Table 1 below.

## 3. Results

**Figure 1:**
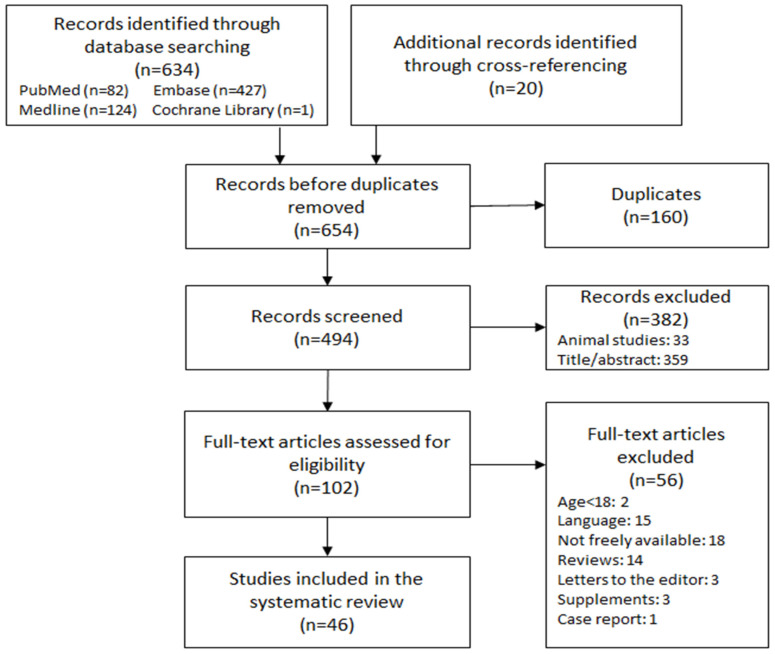
Flow Diagram.

Out of the initial 634 studies identified, 160 duplicates were removed. Following the first selection, 85 studies met the criteria and underwent further evaluation. After the second selection, a total of 46 studies were deemed eligible for inclusion in the qualitative synthesis, as illustrated in Figure 1. The findings from these studies are organized and presented in Table 2, categorized according to the respective screened biomarkers.

### 3.1. I-FABP (Intestinal Fatty Acid-Binding Protein)

A total of 16 studies investigated the use of I-FABP as a biomarker for AMI. Among these studies, 12 focused on serum I-FABP, while three examined the presence of I-FABP in both serum and urine, and one study evaluated the arteriovenous difference (AVD) of I-FABP values. All the studies used ELISA as a method of detection. A total of 2199 patients were included in different studies for the analysis of I-FABP.

The diagnostic value of I-FABP in identifying AMI was compared to various reference groups in different studies, yielding the following findings:

1. Compared to healthy individuals, using a cutoff value of 144.9 pg/mL, I-FABP showed a sensitivity of 71.4% and a specificity of 94.6% [9];
2. In distinguishing acute abdominal pain caused by mesenteric ischemia, a cutoff value of 100 ng/mL yielded a sensitivity of 100% [10] and a specificity of 78.8% and a specificity of 73.8% in another study [11];
3. I-FABP has the potential for diagnosing vascular-related intestinal ischemia but not for nonvascular intestinal ischemia caused by strangulation, hernia, volvulus, or intussusception. A cutoff value of 9.1 ng/mL demonstrated a sensitivity of 83.3% and a specificity of 89.1% [12];
4. Differentiating between strangulated small bowel obstruction (SBO) and simple SBO, a cutoff value of 6.5 ng/mL provided a sensitivity of 71.4% and a specificity of 93.8% [13];
5. I-FABP can serve as a predictor of mortality within 28 days in septic shock patients with intestinal ischemia, using a cutoff value of 19.9 ng/mL, with a sensitivity of 61.5% and a specificity of 86.4% [14];
6. In combination with other indicators, I-FABP at a cutoff value of 9.7 ng/mL exhibited a sensitivity of 70.4% and a specificity of 86% in the diagnosing of pathological pneumatosis intestinalis (PI), which was interpreted as a condition where bowel ischemia requires immediate intervention, irrespective of the underlying pathophysiological processes. In the univariate analysis of this study, portal venous gas was associated with pathologic PI while there was no association in the multivariate analysis. Other radiographic observations, such as free air, ascites, thickened bowel walls, bowel dilation, and stranding, were not associated with pathologic PI [15];
7. In experimentally controlled one-hour ischemia, I-FABP demonstrated a cutoff value of 1.3 ng/mL, a sensitivity of 89%, and a specificity of 100% in detecting irreversible intestinal ischemia–reperfusion damage [16].

Four studies exploring I-FABP also investigated additional biomarkers for AMI, including L-lactate, D-lactate, Interleukin 6 (IL-6), D-dimer, and leukocytes [17,18,19,20]. Combining I-FABP with a cutoff value of 93.03 ng/mL and D-lactate with a cutoff value of 34.28 μg/mL improved the diagnosis of intestinal ischemia (II), with a sensitivity of 76.2% and a specificity of 74.8% [19].

Furthermore, the combination of increased I-FABP, D-lactate, and L-lactate levels showed promise in differentiating acute abdominal pain from proven or likely ischemia (16). I-FABP, at a cutoff value of 90 pg/mL, appeared superior to D-dimer, leucocytes, citrulline, and arginine in the diagnosis of AMI [18,21]. 

Studies assessing urine I-FABP demonstrated higher sensitivity (70–100%) and specificity (78–83%) compared to serum I-FABP, which exhibited sensitivity ranging from 59% to 100% and specificity ranging from 40% to 88% in diagnosing AMI and mechanical SBO. The cutoff values for both urine and serum I-FABP varied from pico- to nanograms [20,22]. Similar trends were observed for urine values of I-FABP, ileal bile acid-binding protein (I-BABP), and urinary L-type fatty acid-binding protein (L-FABP) in relation to adjacent plasma values [23].

In a large multicenter prospective study conducted in France involving 129 patients, including 50 with AMI and a control group of 79 patients with acute abdomen, the combination of I-FABP, D-lactate, and citrulline was measured. Although the biomarker values for all three markers tended to be lower in the AMI population, they were not diagnostically effective in differentiating AMI from an acute abdomen setting [24].

### 3.2. D-Dimer

Eight studies conducted in four different countries involved a total of 963 individuals who were screened. Five different methods of detection were utilized. Some studies focused on using D-dimer alone as a negative predictive value (NPV) for AMI, with cutoff values ranging from 0.3 to 0.9 mg/L, sensitivity ranging from 60% to 100%, and specificity ranging from 36% to 82% [25,26,27]. In certain cases, D-dimer was combined with peritoneal irritation signs to predict reversible ischemia (RI), using a cutoff of 1.65 mg/L, with a sensitivity of 84%, and a specificity of 70%, and bowel necrosis (BN), using a cutoff of 1965 mg/L, with a sensitivity 84% and a specificity of 45.6% [28].

D-dimer may also serve as a predictor for the likelihood of AMI, with even small changes in measured values. For example, a cutoff of 1.0μg FEU/mL demonstrated a sensitivity of 96% and a specificity of 18% in the study Yang et al. [29] in the case of acute bowel ischemia described by Acosta et al. [30]. Confirmation of AMI was achieved with a sensitivity of 84.6% and a specificity of 47.9% among 230 patients presenting with abdominal pain [31]. 

When combined with radiological screening methods, such as CTA, D-dimer showed a cutoff value of 3.17 μg FEU/mL, a sensitivity of 94.7%, and a specificity of 78.6%. This combination proved to be highly effective in the early detection of AMI [32].

### 3.3. L-Lactate

Five studies investigated the use of L-lactate as a biomarker in the diagnosis of AMI, with a combined total of 558 patients. Standard laboratory blood tests were utilized as a screening method across all studies. The results obtained were mixed. In a study involving 91 confirmed AMI patients and 209 lactate measurements, the repeatability of serum lactate measurements had limited value, even in the evaluation of severely ill patients [33]. Similarly, Ambe et al. obtained comparable results in a study of 38 confirmed patients with AMI out of 75 suspected patients with acute abdominal pain, assessing the extent of bowel ischemia [34]. Conde et al. found a correlation between elevated lactate levels and mortality, with a cutoff value of 3.8 mml/L, sensitivity of 81%, and specificity of 76% [35]. Destek et al. compared four biomarkers in patients with AMI:

1. L-lactate cutoff was 3 mmol/L, sensitivity 90.91%, specificity 64.29%;
2. D-dimer cutoff was 1.73 μg/mL FEU, sensitivity 83.33%, specificity 85.71%;
3. CRP cutoff was 19.4 mg/L, sensitivity 92.86%, specificity 69.23%;
4. Neutrophil–lymphocyte ratio (NLR) cutoff was 12.5 × 10^3^/μL, sensitivity 69.23%, and specificity 85.71% [36].

A large multicenter observational study, including 274 patients, of which 137 had AMI, did not find a significant difference between the AMI and control groups [37]. 

### 3.4. Ischemia Modified Albumin (IMA)

Two different studies involving patients suspected of AMI were conducted to evaluate IMA using a cobalt–albumin binding assay (CABA). The case-controlled study on seven patients with AMI in Turkey identified a statistically significant increase in IMA levels among the AMI group compared to the seven controls [38]. Out of 26 patients included in a prospective study conducted in the USA by Polk JD et al., 12 were diagnosed with AMI, and they had significantly higher IMA levels compared to those without intestinal ischemia. The CABA test showed a sensitivity of 100% and a specificity of 85.7% in the preoperative diagnosis of intestinal ischemia [39].

### 3.5. Alpha Glutathione S-Transferase (αGST)

Two prospective studies indicated that αGST may serve as a reliable predictor for the presence or absence of AMI. Delaney CP et al. determined that αGST, with a cutoff value of 4 ng/mL, exhibited a specificity of 85% and a sensitivity of 100% [40]. Similarly, Gearhart SL et al. reported a specificity of 74% and a sensitivity of 97% [41].

### 3.6. Interleukin 6 (IL-6)

In a prospective study involving 46 patients with a confirmed AMI, serum IL-6 levels were significantly elevated when compared to patients with other diagnoses. The study reported a 100% specificity and a sensitivity of 38% at a cutoff value of 20.000 pg/mL [42].

### 3.7. Citrulline

In patients diagnosed with AMI, plasma citrulline displayed 100% specificity, but a lower sensitivity of 39.13%. Regardless of its low sensitivity, Kulu R et al. concluded that, if the citrulline level is >15.82 nmol/mL in patients with acute abdomen or suspected AMI, it is highly likely that there’s no ischemic condition related to the small intestine. Since the difference in plasma citrulline levels was significant (*p* = 0.01) between the AMI and non-AMI groups included in their study, it was suggested that it could be a valuable marker for enhancing the differential diagnosis of patients with acute abdomen, such as acute appendicitis, ulcer perforation, sigmoid colon volvulus, gangrenous cholecystitis, and perforated sigmoid diverticulitis [43].

### 3.8. Procalcitonin (PCT)

Procalcitonin proved to be a useful marker for diagnosing intestinal necrotic damage, determining the extension of necrosis, and predicting the prognosis in patients with mesenteric ischemia. Utilizing time-resolved amplified cryptate emission (TRACE) as a screening method, the predictive thresholds were determined as 2.473 ng/mL for necrosis, 3.884 ng/mL for its extension, and 7.87 ng/mL for mortality [44].

### 3.9. Blood Count

Several parameters within the blood count were examined as potential markers for AMI, including the neutrophil–lymphocyte ratio (NLR), mean platelet volume (MPV), platelet–lymphocyte ratio (PLR), red-cell distribution width (RDW), delta neutrophil index (DNI), and immature granulocytes (IGs).

### 3.10. Neutrophil–Lymphocyte Ratio (NLR)

Multiple studies have illustrated the utility of NLR in the early diagnosis of AMI, with specificity ranging from 72% to 88.71% and sensitivity ranging from 74.14% to 77% [45,46]. Additionally, one study examined the use of NLR for distinguishing AMI from nonvascular bowel necrosis, reporting a specificity of 66.13% and a sensitivity of 50% [47]. 

### 3.11. Mean Platelet Volume (MPV)

According to Türkoğlu A. et al., a high MPV at a cutoff of 8.1 fl supports the diagnosis of acute mesenteric ischemia, with a sensitivity of 83.2%, and a specificity of 80% [48]. However, Degerli V et al., using a higher cutoff of 8.6 fl, obtained lower sensitivity (53%) and specificity (70%) [49]. MPV has also been shown to have prognostic significance in mesenteric ischemia, as nonsurvivors had higher MPV values at presentation compared to survivors [50]. Bilgic IC et al. associated elevated MPV with a worse outcome in patients with AMI [51].

### 3.12. Platelet–Lymphocyte Ratio (PLR)

In a retrospective study, PLR was significantly higher in the AMI group compared to the control group. PLR was identified as an independent predictor of AMI with a sensitivity of 59% and a specificity of 65% [45]. Another study involving 109 participants, categorized into four subgroups based on their PLR values demonstrated a significantly higher 30-day mortality rate in group IV (PLR > 429.3) compared to the other groups (80.8% vs. 46.2%, 66.7%, and 77.8%, *p* = 0.03) [52].

### 3.13. Red-Cell Distribution Width (RDW)

In the diagnosis of AMI, RDW showed a sensitivity of 67.1% and a specificity of 82.1% when using a cutoff value of 13% [46]. However, Kisaoglu et al. concluded that RDW upon admission provides only marginal assistance in diagnosing AMI patients with abdominal pain. They found that, with an RDW cutoff value of 15.04%, the sensitivity, specificity, positive likelihood ratio (+LR), and negative likelihood (-LR) were 40.8%, 81.2%, 2.17, and 0.73, respectively [53].

### 3.14. Delta Neutrophil Index (DNI) and Immature Granulocytes (IGs)

DNI, with a cutoff value of 1,4, exhibited a sensitivity of 70.6% and a specificity of 96.9%. In addition, IG, with a cutoff value of 0.225, illustrated a sensitivity of 74,1% and a specificity of 98.2%. These parameters were found to be more reliable than the routine markers, such as WBC, CRP, and LDH [54].

## 4. Discussion

AMI was shown to have an overall mortality ranging from 50% to 80%, which is why providing a rapid diagnosis and management are of the utmost importance [2]. The proper and timely diagnosis of AMI remains a significant challenge in daily clinical practice since the presenting symptoms are nonspecific or vary widely, and there is no suitable laboratory screening test that can definitively confirm the diagnosis. In clinical practice, there is a significant gap between the appearance of the first clinical signs of the disease, the clinical examination, and the diagnostic definition of the disease, which partly explains the poor treatment outcomes. At the same time, a review of the literature reveals descriptions of the use of individual markers mainly for study purposes and, even with relatively good sensitivity and specificity criteria, no translation into clinical practice. Thus, there remains a gap between the treatment outcome and the therapeutic window that ensures improvement in clinical outcomes. The search for a combination of markers of acute mesenteric ischemia could thus be an appropriate tool for translation into clinical practice. 

Even when there is a clinical suspicion of AMI, confirming the diagnosis has traditionally relied on invasive and time-consuming procedures, such as subtraction angiography or specialized CT techniques, which also require expert radiologic interpretation [55]. Consequently, there is an urgent need to identify serological markers that are clinically useful and unique for detecting intestinal ischemia. Having an accurate marker would be highly advantageous when dealing with complex abdomen cases involving multiple pathologies or when the patient is unresponsive. A marker with sufficient accuracy could help prevent the unnecessary morbidity associated with diagnostic surgical procedures while diagnosing AMI [55,56]. Current guidelines for AMI indicate a lack of reliable markers to definitively determine the presence or absence of ischemic or necrotic bowel. However, elevated levels of L-lactate and D-dimer may offer improved diagnostic accuracy [56]. In this systematic review, 46 studies were included, investigating various markers such as I-FABP, D-dimer, L-lactate, IMA, αGST, IL-6, citrulline, PCT, NLR, MPV, PLR, RDW, DNI, and IGs.

Intestinal fatty acid-binding proteins (I-FABP) are abundant, and also present small cytosolic proteins specific to mature small bowel enterocytes which are located at the villus tip, the area most susceptible to ischemia. When apoptosis occurs in these enterocytes, I-FABP is rapidly released into the bloodstream, indicating compromised cell-membrane integrity [57]. The release of I-FABP has been shown to coincide with the occurrence of mesenteric ischemia in a rat animal model, with serum levels appearing immediately on reperfusion in the ischemia/reperfusion groups and within 15 min in the arterial ligation group [58]. The brief lifespan of plasma I-FABP (11 min) enables the near real-time monitoring of ischemic enterocyte damage. Evidence suggests that I-FABP is highly sensitive and can be detected in the early stages of small bowel ischemia, even when there is only minimal histological damage. Furthermore, the extent of elevation in I-FABP levels corresponds to the severity of mucosal damage, which is influenced by the duration of ischemia and reperfusion [59]. Two studies discovered significantly higher serum levels of I-FABP in a study group of 309 participants compared to healthy controls. While I-FABP cannot differentiate between mesenteric ischemia and intra-abdominal mass, it can distinguish between the aforementioned groups and healthy individuals [9]. In terms of diagnosing AMI, I-FABP levels are considered a more reliable parameter compared to leukocytes and D-dimer elevation [18]. A total of six studies in Japan investigated the usefulness of I-FABP in correlation with AMI. One study identified elevated serum I-FABP in the intensive care unit (ICU) as a predictor for 28-day mortality in adult patients [14]. Three other studies from Japan supported I-FABP as a useful biochemical marker for the accurate diagnosis of AMI [10], identification of patients at risk of small bowel ischemia (SBI) [11], and vascular ischemia [12]. Another study concluded that I-FABP levels are a valuable marker for distinguishing between strangulated small bowel obstruction (SBO), and simple SBO in patients with SBO [13].

A single study explored the potential relationship between the radiological sign pneumatosis intestinalis (PI) and I-FABP values. The findings suggested that elevated serum I-FABP values, in combination with other indicators, could serve as a clinical tool for diagnostic PI [15]. Researchers from the Netherlands conducted three separate studies exploring the utilization of I-FABP at different stages of development. The first experimental study established a cutoff value of 1.3 ng/mL for 60 min of ischemia, demonstrating 100% sensitivity and specificity. The arteriovenous difference in I-FABP levels was used as a sample specimen, indicating the potential value of I-FABP in detecting irreversible intestinal ischemia–reperfusion damage. In addition, systemic I-FABP levels were found to effectively differentiate between mild, reversible IR damage and more extensive irreversible intestinal IR damage [16]. In the remaining two studies, patients with acute abdominal pain were surveyed, with subsequent confirmation of intestinal ischemia (II). One of the two studies investigated additional biomarkers and concluded that I-FABP, L-Lactate, and D-lactate levels were higher in patients with proven or likely ischemia. However, creatine phosphokinase (CPK) did not show a statistically relevant connection with II [17]. 

The last study examined a similar type of the I-FABP molecule in both plasma and urine including I-FABP, L-FABP (urinary L-type fatty acid-binding protein), and I-BABP (ileal bile acid-binding protein) among confirmed II patients. This study discovered that both plasma and urine values of I-FABP and L-FABP, as well as urinary I-BABP levels, could improve early diagnosis of II [23]. Additionally, a study in China concluded that serum I-FABP and D-Lactate could enhance the diagnosis of II in patients with acute abdomen who are at risk [19]. Conversely, C-reactive protein (CRP), CPK, lactate dehydrogenase (LDH), and white blood count (WBC) were neither sensitive nor specific enough. In Canada, both urinary and serum I-FABP were tested, and urinary I-FABP was identified as a noninvasive biomarker with high specificity and sensitivity for AMI [20]. An akin conclusion was reached in a study conducted in the USA, which examined acute mechanical small bowel obstruction and tested both urine and serum I-FABP values [22]. 

It is worth noting that I-FABP concentration is typically low in the plasma of healthy individuals, but it significantly increases within 60 min after ischemia, suggesting that the release of this biomarker correlates with the onset of ischemia [16]. In contrast to these findings, Nuzzo et. al. showed that even when combined with other biomarkers, such as D-Lactate and citrulline, I-FABP was unable to differentiate between patients with early AMI and patients with acute abdomen [24].

D-dimer is a soluble fibrin degradation product that is created as a result of the breakdown of cross-linked fibrin by the enzyme plasmin during the process of fibrinolysis. The specific cleavage of fibrin strands at two sites leads to the release of D-dimer into the bloodstream, where it can be measured as a biomarker of fibrinolytic activity and blood-clot degradation [60]. The timeline for the rise in D-dimer levels in AMI is variable and depends on the extent and duration of ischemia. Generally, D-dimer levels start to increase within a few hours to days after the onset of ischemia. However, they can be also influenced by other factors, including the underlying cause, individual variations, and the presence of comorbidities, as discussed in a multinational study published in WJES in 2022; in the conclusions of the conducted with WJES it has been posted that no accurate biomarkers are available and concluded that there are no relevant markers to guide clinical decisions or serve as prognostic indicators of the disease [56,60]. This means that the sensitivity and specificity of D-dimer depend on the timing of the test, the severity and duration of ischemia, and the underlying cause of AMI. A study conducted in Taiwan on a population of 67 patients with acute abdominal pain using latex turbidimetric immunoassay (LTIA) revealed that D-dimer measurements at a cutoff value of 1.0μg FEU/mL had a high sensitivity of 96% and low specificity of 18%. Although a low D-dimer result slightly decreases the likelihood of AMI, no correlation between the severity of AMI and D-dimer values was observed [29]. A pilot prospective multicenter study in Sweden employing immunofiltration assay (IMFA) found that D-dimer levels indicated the presence of acute bowel ischemia, regardless of the cause, in patients with suspected thromboembolic occlusive (TEO) disease of the superior mesenteric artery (SMA). Larger studies are needed to confirm these findings [30]. Another study in Sweden utilized four different screening methods: quantitative immunofiltration assay (QIMFA), LTIA, turbidimetric inhibition immunoassay (TIBIA), and automated quantitative immune turbidimetric (AUTO-dimer). The study examined several different biomarkers, such as I-FABP, D-dimer, alkaline phosphatase, creatine kinase, lactate dehydrogenase, and αGST. The authors concluded that only D-dimer could be used as an exclusion test for intestinal ischemia, but lacked specificity [27]. A subsequent study in Sweden using QIMFA reaffirmed the usefulness of D-dimer as an exclusion parameter, highlighting its application in a highly selected group with strong clinical suspicion of acute occlusion of the SMA [25]. Similarly, a study in Turkey utilizing ELISA on admission supported the findings of the previous two Swedish studies [26]. In a cross-sectional, single-center study in Turkey, patients with unclear clinical results and D-dimers above 1000 ng/mL, combined with atrial fibrillation (AF), were recommended for further evaluation for AMI. The study acknowledged the limitation of using LTIA as a screening method as a limitation compared to ELISA [31]. The last study about D-dimer in Turkey employing QIMFA demonstrated that D-dimer, in combination with CTA, is a highly sensitive test for the early diagnosis of AMI [32]. In a study conducted in China, the population was divided into reversible ischemia (RI) and bowel necrosis (BN). Both subgroups exhibited a sensitivity of 84%, with bowel necrosis showing a lower specificity of (45.6%) compared to reversible ischemia (70%). Combining D-dimer with peritoneal irritational signals could help achieve a reliable negative predictive value [28]. Thus, D-dimer can be considered as a biomarker to rule out the presence of AMI in suspected patients [56].

L-lactate is an isomer of lactate that is formed out of pyruvic acid by the enzymes LDH as an end product of anaerobic glycolysis. Tissue hypoperfusion decreases lactate metabolism in the liver or kidney, and malignancy and diabetic ketoacidosis are some of the conditions that lead to increased serum L-lactate levels [61]. Research in Germany found no linear association between serum lactate and the extent of bowel ischemia, albeit this study has considerable selection bias, only managing patients within 24 h of the presentation of symptoms [34]. Similar findings in Switzerland were attained, serum lactate is of limited value even when measured repeatedly in the evaluation of severely ill patients with AMI. Nonetheless, the measurements were obtained at the discretion of the physician, resulting in a diverse variety of timings of the blood tests [33]. A retrospective study in Turkey compared L-lactate, D-dimer, CRP, and NLR. From all four markers, CRP proved to be an easily accessible, inexpensive, effective, and valuable addition to the screening of various subtypes of AMI. L-lactate was found to not have any diagnostic value in the etiological types of AMI [36] or have any correlation with AMI [37]; only Conde et al. found that it is a useful diagnostic tool for predicting mortality [35].

Ischemia-modified albumin (IMA) was shown to be a sensitive marker in some acute ischemic conditions, such as cerebral, myocardial, or pulmonary infarct [62,63,64]. The findings of Gunduz A et al. indicate that IMA could be used for the early diagnosis of AMI. However, a substantial limitation of this study is the small sample size [38]. A pilot study in the USA concluded that CABA is practical in the stratification of II. This study, except for a small population, had a lack of a validated observational method for determining the degree of ischemia present during laparotomy [39]. Further prospective research with a larger sample size compared to a control group needs to be carried out to determine the utility of this biomarker.

Alpha glutathione S-transferase (αGST) is an enzyme involved in the detoxification process by facilitating the conjugation of reduced glutathione with toxic compounds, making them more water soluble for elimination via feces or urine. αGST is present in the liver and kidney, as well as intestine [40,65,66]. It is predominantly located in the cells of the intestinal mucosa and is released when the cell membrane is damaged [67]. In both studies included in this systematic review, αGST was shown to have a high specificity (97% and 100%), suggesting its potential to reliably exclude the presence of AMI when the αGST levels are below the cutoff value of 4 ng/mL [19,23]. Evennett et al. confirmed that αGST could be beneficial in low-prevalence settings, such as general surgical patients with acute abdominal pain and individuals with severe acute pancreatitis, due to its high pooled specificity of 0.85 [64]. 

Interleukin 6 (IL-6) is a cytokine that plays a role in a variety of biological activities, exhibiting both anti-inflammatory and proinflammatory effects [68]. Sutherland et al. demonstrated that serum interleukin-6 levels were significantly elevated in patients with confirmed acute intestinal ischemia compared to other diagnoses (15.778 ± 21.349 pg/mL vs. 2.844 ± 5.625 pg/mL, *p* = 0.01) [42]. 

Citrulline is an α-amino acid primarily synthesized by small bowel enterocytes from glutamine [69,70]. After being released from enterocytes into the portal circulation, it enters the systematic circulation and undergoes breakdown in the kidneys, where it is converted back into arginine and released into the plasma [71]. This establishes a correlation between enterocyte mass, gut synthesis, and renal metabolism [72,73]. Citrulline is recognized as a marker for acute and chronic intestinal insufficiency [72]. Measuring plasma citrulline using ion-exchange chromatography may augment our ability to differentially diagnose patients with acute abdominal conditions. Although most studies exploring the association between AMI and citrulline have been conducted on animals and are excluded from this systematic review, the human studies available present mixed results [21,24,43].

Procalcitonin (PCT) is a 116 amino-acid precursor of calcitonin, secreted by liver parenchyma and other sources during severe bacterial infection [74]. Cosse et al. evaluated PCT as a diagnostic marker for intestinal necrotic damages, their extent, and prognosis using a gray-zone approach in a population with ischemic colitis and mesenteric infarction. The study confirmed that PCT could be utilized as an indicator of necrosis, predicting patient mortality and the extent of damage [44].

Intestinal ischemia leads to the disruption of the intestinal barrier and the translocation of endotoxins, which trigger an inflammatory response characterized by complement activation, endothelial activation, neutrophil sequestration, and the release of proinflammatory mediators into the bloodstream [64,75]. In AMI patients, the neutrophils increase and accumulate in the damaged tissues due to the activation of the complement system [75], while lymphocytes decrease due to the excessive secretion of cortisol, which is a physiological response to stress caused by the underlying pathology [76]. On the other hand, platelets modulate leukocyte function but also contribute to tissue injury caused by ischemia by producing proinflammatory mediators, such as platelet factor-4, leukotriene, thromboxane, platelet-derived growth factor, and serotonin. These mediators stimulate the proliferation of megakaryocytes, resulting in relative thrombocytosis [77]. The inflammatory process and the release of cytokines can also cause damage to the membranes of mature erythrocytes, leading to an increase in the RDW value. However, the increased erythrocyte turnover in anemia can also result in an elevated RDW, which is important to rule out before establishing an AMI diagnosis [78]. This is why different hematological laboratory markers, such as NLR, MPV, PLR, RDW, DNI, and IG were investigated in relation to AMI. Three studies examined the association between NRL and AMI. Tanrikulu et al. found that NLR can differentiate between nonvascular bowel necrosis (NVBN) and AMI, making it a useful adjunct to clinical examination [47]. Aktimur et al. explored combinations of NLR, RDW, white blood cells (WBC), and MPV. NLR values >9.9, along with RDW and other clinical assessments, were found to be helpful in the diagnosis of AMI, especially when advanced imaging modalities or expert radiological interpretation are not available. However, this study lacked access to certain data, such as laparotomy time and time from the start of pain to admission [46]. Toptas et al. also investigated a combination of classical laboratory biomarkers including NLR, PLR, and CRP. NLR was an independent predictor of AMI compared to sex-matched healthy control groups, similar to PLR. A limitation of this study was the lack of histopathologic confirmation for patients with AMI to determine the exact cause of thrombus formation [45]. Augene E et al. determined that PLR, but not NLR, was a predictive factor of 30-day mortality in patients with acute mesenteric ischemia. However, future research with a larger number of patients is needed to confirm these results [52]. High MPV values in routine hemograms supported the diagnosis of AMI in patients admitted to the hospital with acute abdominal nonspecific pain (47) and were associated with worse outcomes among AMI patients [51]. Degerli V et al. noted that MPV can only serve as an indicator of AMI when patients do not have concomitant diseases. However, this retrospective study had limited data available, and patient medications were not taken into account [49]. Antiplatelet drugs and lipid-lowering drugs affect platelet size [79,80]. Altiltoprak et al. retrospectively examined 30 operated patients with AMI, dividing them into survivors and nonsurvivors, and found that MPV values at presentation were higher in nonsurvivors [50]. Kisaoglu et al. investigated RDW in terms of sensitivity and specificity for AMI diagnosis in patients with abdominal pain. RDW showed limited assistance in diagnosing AMI. Additionally, this study compared groups with conditions such as chronic heart failure, arterial hypertension, and AF, which lack similarity and are also risk factors for AMI, potentially leading to significantly higher RDW values in AMI patients [53]. Durak D et al. demonstrated that IG and DNI have the potential to serve as markers for evaluating intestinal necrosis in mesenteric ischemia, without the need for additional time or expenses for diagnosis, as they can be measured within a complete blood count [54].

## 5. Limitations

Small sample sizes, observed in some of the included studies, could lead to an overestimation of the true diagnostic accuracy of biomarkers for acute intestinal ischemia. Additionally, different studies employ varying cutoff values, ranging from picometers to nanometers, making it challenging to determine the optimal cutoff value. The use of different screening methods for the same markers further complicates the standardization of identifying the most effective marker. Moreover, the reference tests for diagnosing AMI differ across studies. While histopathological examination serves as the golden standard reference, its complexity in acquiring pathological specimens for certain diseases is often overlooked. Some included studies relied on radiological examination or mixed reference standards to diagnose acute intestinal ischemia, potentially resulting in an overestimation of diagnostic accuracy. Furthermore, many less-known biomarkers have predominantly been studied on animal subjects, leading to a limited number of studies available for a sensible comparison with our systematic review. A common challenge in systematic reviews is the presence of language barriers that may result in excluding publications that do not meet the inclusion criteria. In our study, due to limited resources and team capacity, we only included studies published in English, Slovenian, Serbo-Croatian, or Macedonian, which could introduce language bias. Furthermore, the bibliography exclusively comprises freely available articles.

## 6. Conclusions

This systematic review aimed to gather available information on potential biomarkers for diagnosing AMI. Various biomarkers exhibited different levels of accuracy in detecting the condition. Among them, I-FABP and D-dimer emerged as the most extensively researched and valuable biomarkers for AMI diagnosis. Other biomarkers such as IMA, αGST, IL-6, and citrulline demonstrated potential usefulness based on individual studies, but further research with larger sample sizes and appropriate controls is needed to reduce bias. Notably, several studies highlighted the potential utility, wide availability, and cost effectiveness of NLR, PLR, MPV, IG, DNI, and RDW as diagnostic tools. Given the challenge of identifying a single specific biomarker, future researchers should focus on examining and calculating the collective diagnostic impact of these potential biomarkers. Such investigations will aid in developing AMI laboratory panels comprising a combination of biomarkers that reflect various aspects of intestinal viability.

## Figures and Tables

**Table 1:** Eligibility criteria for study selection.

Inclusion Criteria	Exclusion Criteria
- Adult patients diagnosed with acute mesenteric ischemia, intestinal ischemia, small bowel obstruction, bowel necrosis, reversible/irreversible bowel ischemia, a mesenteric infarction. - English, Slovenian, Serbo-Croatian, Macedonian language - Freely available studies	- Patients < 18 years - Studies in other languages - Animal studies - Supplements - Reviews - Letters to the editor - Case studies

**Table 2:** Overview of selected studies and their corresponding biomarkers.

Studied Marker	Country	Study Population (n)	Design	Sample Tissue	Method of Detection	Cutoff	Sensitivity (Sen)	Specificity (Spe)	Major Findings	Reference Test	LR
I-FABP	Turkey	AAP (171), from which 7 with AMI, Control group: 130 Total: 301	Cohort, Prospective, Single Center	Blood	ELISA	AMI: 144.9 pg/mL	AMI: 71.4%	AMI: 94.6%	Serum I-FABPs were significantly higher when compared to those of healthy volunteers.	PE, US, CT	[9]
I-FABP	Japan	AAP (61), from which 5 with MI. Control group: 35 Total: 96	Cohort, Retrospective, Multicenter	Blood	ELISA	MI: 100 ng/mL	MI: 100%	MI: /	I-FABP is a useful biochemical marker for the accurate diagnosis of mesenteric infarction	Laboratory findings, Histopathologic findings	[10]
I-FABP	Japan	AAP (361) from which 52 with SBI. Control group: 119 Total: 361	Cohort, Prospective, Multicenter trial	Blood	ELISA	/	78.8%	73.8%	Serum I-FABP is potentially useful for identification of patients who are at risk of small bowel ischemia.	Laparotomy	[11]
I-FABP	Japan	Suspect for AMI (208) from which 66 had II. Control group: 122 Total: 208	Cohort, Prospective, Single Center	Blood	ELISA	9.1 ng/mL	83.3%	89.1%	I-FABP shows promise for detecting vascular ischemia.	PE, Laboratory findings, CT, Laparotomy, Histopathologic findings	[12]
I-FABP	Japan	AAP (287) from which 21 diagnosed with SBO Control group: 16 Total: 287	Cohort Retrospective Single Center	Blood	ELISA	6.5 ng/mL	71.4%	93.8%	I-FABP levels are a useful marker for discriminating between strangulated SBO and simple SBO in patients with SBO.	PE, CT, Surgery	[13]
I-FABP	Japan	Septic shock patients (57) from which 16 with intestinal ischemia Total: 57	Cohort, Prospective, Single Center	Blood	ELISA	19.9 ng/mL	61.5%	86.4%	Elevated I-FABP at ICU admission can serve as a 28-day mortality predictor in adult patients.	Laboratory findings, CT, Surgery	[14]
I-FABP	Japan	Suspected PI (70), from which 27 confirmed Total: 70	Cohort, Prospective, Single center	Blood	ELISA	PI: 9.7 ng/mL	PI: 70.4%	PI: 86%	High I-FABP value, in combination with other indicators, can be clinically useful for pathologic PI.	PE, Laboratory Findings, CT, Surgery	[15]
I-FABP	NL	Experimental IR (27). Control group: 5 Total: 32	Cohort, Experimental, Cross Sectional, Single center	AVD	ELISA	60I: 1.3 ng/mL	60I: 90%	60I: 100%	Systemic I-FABP levels appear valuable in detecting irreversible intestinal ischemia-reperfusion damage.	Histopathologic findings, immunohistochemistry	[16]
I-FABP L-Lactate CPK D-Lactate	NL	AAP (120) from which 23 with II. Control group: 21 Total: 120	Cohort Prospective	Blood	ELISA SLBT KSM	/ 2.2 mmol/L / /	/ 78% / /	/ 48% / /	I-FABP, L-Lactate, and D-Lactate levels were higher in patients with proven or likely ischemia.	PE, Laboratory findings CT, Laparotomy, Histopathologic findings	[17]
I-FABP D-dimer Leu	Turkey	AAP (57), from which 30 with AMI. Control Group: 20 Total: 77	Cohort, Prospective, Single Center	Blood	ELISA	AMI: 90 pg/mL 130 μg/mL 11,042 mm^3^	AMI: 90% 93% 90%	AMI: 100% 100% 100%	I-FABP level is a more reliable parameter for diagnosis of AMI compared to Leu and D-dimer elevation.	Histopathological findings, Surgery	[18]
I-FABP D-lactate CPK CRP LDH WBC	China	AAP (272), from which 39 with II. Control group: 37 Total: 309	Cohort, Prospective, Singe Center	Blood	ELISA	93.03 ng/mL 34.28 μg/mL / / / /	II: 76.2% 66.7% 31.7% 68.9% 61.6% 61.6%	II: 74.8% 85.9% 79.2% 34.2% 77.3% 36.5%	Serum I-FABP and D-lactate can improve the diagnosis of II in patients with acute abdomen who are at risk.	Histopathologic findings, CT angiography	[19]
I-FABP IL-6, L-Lactate	Canada	AMI (13) Control group: 5 Total: 18	Cohort, Prospective, Single Center	Urine Blood	ELISA	U: 2.52 ng/mL B: 0.04 ng/mL 0.04 ng/mL 3.20 ng/mL	U: 91.7% B: 92.3% 100% 58.3%	U: 80% B: 40% 60% 75%	Urine I-FABP is a noninvasive biomarker with high specificity and sensitivity for AMI	PE, Laboratory findings, Histopathologic findings, Surgery	[20]
I-FABP Citrulline Arginine	France	NOMI (33) patients. Control group: 28 Total: 61	Prospective, observational Multicenter	Blood	ELISA IE CHR IE CHR	3114 pg/mL / /	70% / /	85% / /	Elevated I-FABP was significantly associated with intestinal necrosis.	PE, Laboratory Findings, CT, GI Endoscopy, Laparatomy.	[21]
I-FABP	USA	AMSBO (21), from which 14 underwent laparotomy Total: 21	Cohort, Prospective, Single Center	Urine Blood	ELISA	/	U: 100% B: 100%	U: 83% B: 78%	I-FABP is a sensitive marker for ischemia in mechanical small bowel obstruction.	PE, Laboratory findings Laparotomy, CT	[22]
I-FABP L-FABP I-BABP	NL	AAP (52) from which 22 with II. Control group: 24 Total: 52	Cohort, Prospective, Single center	Urine Blood	ELISA	U: 551 pg/mL B: 268 pg/mL U: 180 ng/mL B: 78 ng/mL U: 5 ng/mL B: 7 ng/mL	U: 90% B: 68% U: 80% B: 59% U: 70% B: 64%	U: 89% B: 71% U: 78% B: 88% U: 89% B: 64%	Plasma and urine, I-FABP and L-FABP, and urinary I-BABP levels can improve early diagnosis of II.	Laparotomy, Histopathologic findings, Surgery, Autopsy	[23]
I-FABP D-lactate Citrulline	France	AMI (50) Control group: 79 Total: 129	Cohort, prospective, Multicenter	Blood	ELISA KSM IE CHR	974 ng/L 0.012 mmol/L 16.6 μmol/L	15% 98% 56%	95% 17% 84%	I-FABP, D-lactate, and Citrulline failed to differentiate AMI from acute abdominal controls.	PE, Laboratory Findings, CT, IRA, Laparotomy, Histopathologic findings	[24]
D-dimer	Sweden	AAP (101), from which 9 with acute SMA occlusion. Control group (92) Total: 101	Cohort, Prospective, Single center	Blood	QIMFA	0.3 mg/L	100%	36%	D-dimer testing may be useful for the exclusion of patients with suspected acute SMA occlusion	PE, Laboratory findings, CT, Laparotomy, Histopathologic findings	[25]
D-dimer	Turkey	Nontraumatic acute abdominal gastrointestinal disorders (159), from which 33 had II. Control group (166) Total: 159	Cohort Prospective, Single center	Blood	ELISA	/	85%	41%	An elevated D-dimer level on admission had a high sensitivity for identifying patients with intestinal ischemia, although it had a low specificity.	Laboratory findings, Laparotomy	[26]
D-dimer	Sweden	AAP (71), from which 10 with II. Control group (61) Total: 71	Cohort, Prospective, Single center	Blood	1.QIMFA 2.LTIA 3.TINIA 4.AUTO Dimer	0.3 mg/L 0.6 mg/L 0.9 mg/L	100% 80% 60%	44% 75% 82%	D-dimer may be used as an exclusion test for intestinal ischemia but lacks specificity.	PE, Laboratory findings, CT, Laparotomy, Histopathologic findings	[27]
D-dimer	China	Suspected for BN (274), from which 99 with necrosis, Control group (158) Total: 274	Cohort, Retrospective, Single Center	Blood	QIMFA	RI: 1.65 mg/L BN: 1965 mg/L	84% 84%	70% 45.6%	Combination of D-dimer and peritoneal irritational signals could help generate reliable NPV.	PE, Laboratory findings, CT, Histopathology findings, Surgery	[28]
D-dimer	Taiwan	AAP (67) from which 23 with AMI. Control group (44) Total: 67	Cohort, Prospective, Single Center	Blood	LTIA	1.0 μg FEU/mL	96%	18%	Measurement of D-dimer levels can be of value for a small decrease in the likelihood of AMI, when the result is low.	PE, Laboratory findings, CT, Laparotomy, Histopathologic findings	[29]
D-dimer	Sweden	Suspect for acute bowel ischemia (14), from which 6 with AMI. Control group (8) Total: 14	Pilot, Prospective, Multicenter	Blood	IMFA	/	/	/	In patients with suspected TEO of the SMA, D-dimer indicates presence of acute bowel ischemia, whatever the cause.	Laparotomy	[30]
D-dimer	Turkey	Suspected for AMI (230) from which 23 with AMI confirmed. Control group (203) Total: 230	Cohort, Cross-sectional, Single center	Blood	LTIA	/	AMI: 84.6%	AMI: 47.9%	Patients suspected of having AMI with unclear clinical results and patients with D-dimer levels above 1000 ng/mL and AF should undergo further evaluation.	PE, Laboratory findings, CT,	[31]
D-dimer	Turkey	Suspected for AMI (47), from which 28 had AMI. Control group (19) Total: 47	Cohort, Prospective, Single Center	Blood	QIMFA	3.17 μg FEU/mL	94.7%	78.6%	D-dimer in combination with CTA is useful, highly sensitive test for early diagnosis of AMI	PE, Laboratory findings, CT, Laparotomy	[32]
L-lactate	CH	Confirmed AMI (91) in which 209 lactate measurements were made Total: 91	Retrospective, Single Center	Blood	SLBT	/	/	/	Serum lactate is of limited value, even when measured repeatedly in evaluation of severely ill patients with AMI	PE, Laboratory findings, Medical History, Histopathology findings, Surgery	[33]
L-lactate	DE	AAP (75), from which 38 with AMI. Total: 75	Cohort, Retrospective, Single Center	Blood	SLBT	/	/	/	A linear association between serum lactate and extent of bowel ischemia could not be established.	PE, Laboratory findings, US, RTG, CT, Laparotomy	[34]
L-lactate	CO	Confirmed for AMI (74) Total: 74	Retrospective, Cross sectional, Single Center	Blood	SLBT	3.8 mmol/L	81%	76%	A useful prognostic tool in terms of mortality in patients with AMI.	PE, Laboratory findings, Laparotomy	[35]
L- lactate D-Dimer CRP NLR	Turkey	Confirmed for AMI (44) Total: 44	Retrospective, Single Center	Blood	SLBT	L-Lactate: 3 mmol/L D-Dimer: 1.73 μg/mL FEU CRP: 19.4 mg/L NLR: 12.5 × 10^3^/μL	L-Lactate: 90.91% D-Dimer: 83.33% CRP: 92.86% NLR: 69.23%	L-Lactate: 64.29% D-Dimer: 85.71% CRP: 69.23% NLR: 85.71%	CRP is an easily accessible, inexpensive, effective, and valuable addition to screening of various subtypes of AMI.	PE, Laboratory findings, Laparotomy	[36]
L-lactate	France	AMI (137) Control group: 137 Total: 274	Ancillary, retrospective, observational, Multicenter	Blood	SLBT	5.1 mmol/L	64%	87%	No link was observed between lactate levels and the diagnosis or outcomes of AMI.	PE, Laboratory Findings, SAPS II score, Laparatomy	[37]
IMA	Turkey	7 patients with confirmed AMI. Control group: 7 Total: 14	Case Control, Single Center	Blood	CABA	/	/	/	Statistically significant increase of IMA values was seen in AMI population	Laboratory Findings	[38]
IMA	USA	Confirmed II (12), out of 26 patients for explorative laparotomy Control group: 14 Total: 26	Pilot, Prospective, Single Center	Blood	CABA	/	100%	85.7%	The CABA tool can be a useful tool for clinicians in the risk stratification of II.	PE, Laboratory findings, Surgery	[39]
αGST	Ireland	AAP (26) from which 12 with AMI. Control group: 14 Total: 26	Cohort, Prospective, Single Center	Blood	ELISA	4 ng/mL	100%	86%	αGST may reliably predict the presence or absence of AMI.	PE, Laboratory findings, Radiological findings, Surgery, Autopsy	[40]
αGST	USA	Suspected for AMI (58), from which 35 were confirmed. Control group: 23 Total: 58	Cohort, Prospective, Single Center	Blood	ELISA	/	97%	74%	A normal αGST and WBC may exclude presence of AMI.	PE, Laboratory findings, Radiological findings, Surgery	[41]
IL-6	Canada	Confirmed AMI patients compared with other diagnoses Total: 46	Cohort, Prospective, Single Center	Blood	ELISA	20.000 pg/mL	38%	100%	Serum IL-6 may prove useful in diagnosing patients with AMI	PE, Laboratory findings, Surgery	[42]
Citrulline D-dimer L-lactate	Turkey	AAP (48), from which 23 with AMI. Control group: 25 Total: 48	Cohort, Prospective, Single Center	Blood	IE CHR / /	15.82 nmol/L 2126 μg/L 3.1 mmol/L	39.13% 78.26% 39.13%	100% 80% 96%	The measurement of plasma citrulline may improve our ability in differential diagnosis of patients with acute abdomen.	PE, Laboratory findings, Surgery	[43]
PCT	France	MI or IC (128) with either, from which 34 with ischemic damages, 94 with ND. Total: 128	Cohort, Retrospective, Multicenter	Blood	TRACE	ND: 2.473 ng/mL ETD: 3.884 ng/mL Mo: 7.87 ng/mL	ND: 94.6% ETD: 76.3% Mo: 72%	ND: 68% ETD: 84.2% Mo: 79.6%	PCT could be used as a marker for necrosis, especially in case of extended damage, and reflects a patient’s prognosis.	PE, Laboratory findings, Surgery, Histopathology findings	[44]
NLR PLR CRP	Turkey	AMI (46) Control group: 46 Total: 92	Cohort, Retrospective, Single Center	Blood	SLBT	4.6 / /	77% / /	72% / /	Increased NLR and PLR were independent predictors of AMI.	PE, Laboratory findings, Surgery	[45]
NLR RDW WBC MPV	Turkey	Underwent laparotomy or bowel resection (70) for AMI. Control group: 123 Total: 193	Cohort, Retrospective, Single Center	Blood	SLBT	9.9 13% 14.4 /μL 10.5 fL	74.3% 67.1% 57.1% 60%	82.9% 82.1% 69.3% 71.5%	High NLR value seems to be a valuable diagnostic marker of AMI.	PE, Laboratory findings, Surgery, Histopathology findings	[46]
NLR WBC CRP RDW MPV	Turkey	Abdominal pain (182), from which 58 AMI, 62 NVBN. Control group: 62 Total: 182	Cohort Retrospective, Cross-sectional Multicenter	Blood	SLBT	AMI-CG: 5.21 AMI-NVBN: 7.85	AMI-CG: 74.14% AMI-NVBN: 50.00%	AMI-CG: 88.71% AMI-NVBN: 66.13%	NLR aids in the diagnosis of AMI and can be used to distinguish from NVBN.	PE, Laboratory findings, Surgery	[47]
MPV	Turkey	AMI (95) Control group: 90 Total: 185	Cohort, Retrospective, Single Center	Blood	SLBT	8.1 fL	83%	80%	High mean platelet volume values support the diagnosis of AMI.	PE, Laboratory findings, Surgery	[48]
MPV	Turkey	AMI (41) Control group: 82 Total: 123	Cohort, Case control, Retrospective, Single Center	Blood	SLBT	8.6 fL	70%	53%	MPV may be used as an indicator of AMI only if the patient has no concomitant diseases.	PE, Laboratory findings, Radiological findings, Surgery	[49]
MPV	Turkey	AMI (15), comparing survival versus dead Control group: 15 Total: 30	Cohort, Retrospective, Single Center	Blood	SLBT	/	/	/	MPV can be beneficial in predicting patients with poor prognosis and in the planning of reoperations.	PE, Laboratory findings, Surgery	[50]
MPV	Turkey	AMI (61), comparing survival versus dead Control group: 26 Total: 61	Cohort, Retrospective, Single Center	Blood	SLBT	/	60%	73.08%	Elevated MPV is associated with a worse outcome in patients with AMI.	PE, Laboratory findings, Surgery	[51]
PLR NLR	France	AMI (106) Total: 106	Cohort, Retrospective, Single Center	Blood	SLBT	/	/	/	PLR, but not NLR, is a predictive factor of 30-day mortality in patients with AMI.	PE, Laboratory findings, CT, Surgery	[52]
RDW WBC LDH BUN	Turkey	AAP (169), from which AMI (49) Control group: 110 Total: 159	Cohort, Case-control, Retrospective, Single Center	Blood	SLBT	15.04% 12,900 /μL 299.5 U/L 19.9 mg/dL	40.8% 71.4% 87.8% 71.4%	81.2% 81.2% 76% 60.4%	RDW on admission is of marginal help to diagnose AMI patients with abdominal pain	PE, Laboratory findings, CT, Surgery	[53]
DNI IG WBC LDH CRP	Turkey	AMI (85) Control Group: 163 Total: 248	Cohort, Retrospective, Single Center	Blood	SLBT	1.4 0.225 8.59 214 12.6	70.6 74.1 60 70.6 64.7	96.9 98.2 62.6 60.7 64.4	IG and DNI are reliable markers that do not require additional expenses	PE, Laboratory findings, Surgery	[54]

## Abbreviations

60I	60 min lasting ischemia
AAP	acute abdominal pain
AF	atrial fibrillation
αGST	alpha glutathione S-transferase
AMAE	acute mesenteric arterial embolism
AMSBO	acute mechanical small bowel obstruction
AMAT	acute mesenteric arterial thrombosis
AMI	acute mesenteric ischemia
AUTO-Dimer	automated quantitative immuno-turbidimetric D-dimer
AVD	arteriovenous difference
B	blood
BN	bowel necrosis
CABA	cobalt–albumin binding assay
CG	Control Group
CH	Switzerland
CK	creatinine kinase
CO	Colombia
CPK	creatinine phosphokinase
CTA	computed tomography angiography
DE	Germany
DNI	delta neutrophil index
ETD	extended tissue damages
FEU	fibrinogen equivalent units
GI	Gastro-intestinal
I-BABP	ileal bile acid-binding protein
IC	ischemic colitis
IE CHR	ion-exchange chromatography
I-FABP	intestinal fatty acid-binding protein
IGs	immature granulocytes
II	intestinal ischemia
IL	interleukin
IL-6	interleukin 6
IMA	ischemia modified albumin
IMFA	immunofiltration assay
IR	ischemia reperfusion
IRA	interventional radiology approach
KSM	kinetic spectrophotometric method
LDH	lactate dehydrogenase
LEU	leukocyte
LR	literature reference
LTIA	latex turbidimetric immunoassay
Lab.	laboratory
L-FABP	urinary L-type fatty acid-binding protein
MI	mesenteric infarction
MO	mortality
MOF	multiple organ failure
MPV	mean platelet volume
MVT	mesenteric venous thrombosis
ND	necrotic damages
NL	Netherlands
NLR	Neutrophil–lymphocyte ratio
NOMI	nonocclusive mesenteric ischemia
NPV	negative predictive value
NVBN	nonvascular bowel necrosis
PCT	procalcitonin
PE	physical examination
PI	pneumatosis intestinalis
PLR	platelet–lymphocyte ratio
QIMFA	quantitative immunofiltration assay
RDW	red-cell distribution width
RI	reversible ischemia
SAPS II	Simplified Acute Physiology Score
SBI	small bowel ischemia
SBO	small bowel obstruction
SLBT	standardized laboratory blood test
SMA	superior mesenteric artery
TEO	thromboembolic occlusion disease
TINIA	Turbidimetric inhibition immunoassay
TRACE	time-resolved amplified cryptate emission
U	urine
US	ultrasound
USA	United States of America
WBC	white blood count
